# A Small Object Detection Algorithm Based on Modulated Deformable Convolution and Large Kernel Convolution

**DOI:** 10.1155/2023/2506274

**Published:** 2023-01-24

**Authors:** Hongxia Yu, Lijun Yun, Zaiqing Chen, Feiyan Cheng, Chunjie Zhang

**Affiliations:** ^1^College of Information, Yunnan Normal University, Kunming 650500, Yunnan, China; ^2^Yunnan Province Key Laboratory of Opto-Electronic Information Technology, Yunnan Normal University, Kunming, Yunnan 650500, China

## Abstract

Object detection is one of the most critical areas in computer vision, and it plays an essential role in a variety of practice scenarios. However, small object detection has always been a key and difficult problem in the field of object detection. Therefore, considering the balance between the effectiveness and efficiency of the small object detection algorithm, this study proposes an improved YOLOX detection algorithm (BGD-YOLOX) to improve the detection effect of small objects. We present the BigGhost module, which combines the Ghost model with a modulated deformable convolution to optimize the YOLOX for greater accuracy. At the same time, it can reduce the inference time by reducing the number of parameters and the amount of computation. The experimental results show that BGD-YOLOX has a higher average accuracy rate in terms of small target detection, with mAP0.5 up to 88.3% and mAP0.95 up to 56.7%, which surpasses the most advanced object detection algorithms such as EfficientDet, CenterNet, and YOLOv4.

## 1. Introduction

Object detection has made remarkable progress in recent years due to the development of deep learning [1–8]. However, it is still a puzzle to detect small objects in the field of object detection [9]. Small objects are objects with pixel areas of less than 32 × 32 pixels, defined in COCO, a common dataset in object detection. There are three main difficulties with small object detection.

First, small objects cover a smaller area and therefore have fewer useful semantic features. Second, the number of small target instances is lower, potentially making the object detection model pay more attention to detecting large targets. Third, the anchors are difficult to match. For the anchor-based method, due to the small object's ground truth being very small, if the anchor is improperly set, the IoU between the small object's ground truth and the anchor is too low. It may cause the network to see the anchors as negative samples.

Nowadays, object detection algorithms mainly improve the effect of small object detection through multiscale detection [10, 11], multiscale feature fusion [12, 13], data augmentation [9, 14], and resolution enhancement [15–17]. Lin et al. [13] proposed feature pyramid networks (FPNs). It fuses high-dimensional feature maps and low-dimensional feature maps by upsampling. It increases the resolution of the feature map, obtains more useful information about small objects, and improves the detection performance of small targets. PANet [16] added a path after the FPN to convey the positioning features bottom-up, forming a bidirectional feature pyramid. EfficientDet [18] proposed a bidirectional feature pyramid network (BiFPN), which allows simple and fast multiscale feature fusion. Mostly, the input resolutions vary and contribute differently to the output feature maps. Therefore, BiFPN introduces learnable weights to learn the importance of different input features, at the same time, repeatedly applying top-down and bottom-up multiscale feature fusion. Kisantal et al. [9] improved the detection effect of small objects through data augmentation. During training, oversampling the images with small objects solves the problem of having fewer images with small objects, and using the copy and paste strategy increases the number of small objects. SSD [19] improved the detection ability of small targets through multiscale detection. Lower-dimensional feature maps are applied to detect smaller targets, and higher-dimensional feature maps are used to detect larger targets. SOD-MTGA [17] obtained the subgraph containing small targets by the trained detector and then uses the generator to generate the corresponding high-definitional image, and the discriminator is responsible for determining the authenticity of the generated image and predicting the category and location of small targets.

These strategies can improve the detection performance of small objects to a certain extent. Nonetheless, due to the fact that the number of small object samples is small and the information on small object images is limited, there will still be misses and false detections.

The current one-stage object detection algorithms balance speed and accuracy well. Among them, the YOLO series algorithm is one of the most famous series, including YOLOv1 [20], YOLOv2 [21], YOLOv3 [3], YOLOv4 [1], YOLOv5 [22], and YOLOX [23]. In these algorithms, YOLOX is quite different from other algorithms in the YOLO family. YOLOX skillfully combines some fresh algorithm improvement strategies, such as anchor-free mechanism, decoupled head, and label assignment, with YOLO's network structure, thus greatly improving its performance. In this paper, an improved YOLOX model (BGD-YOLOX) is proposed, which uses some of the latest algorithmic strategies to improve the performance of the network to detect small objects.

## 2. Related Work

The anchor-based method is still mainstream in object detection [1, 3, 4, 8, 21, 22, 24, 25], which predefines some anchors and generates bounding boxes based on these anchors. Many one-stage object detection algorithms such as YOLOv2 [21], YOLOv3 [3], YOLOv4 [1], RetinaNet [7], and EfficientDet [18] are all anchor-based methods. Two-stage object detection algorithms including Faster R-CNN [24], FPN [13], Cascade R-CNN [2], and TridentNet [5] are all anchor-based algorithms.

Moreover, the anchor-free method has attracted increasing attention. In recent years, increasingly anchor-free algorithms have been proposed. The anchor-free method has the following two types:

- Keypoint-based algorithms first detect the upper left and lower right corners of the object and then output the predictions through corner matching and corner position offset, including CenterNet [26], CornerNet-Lite [27], CornerNet [28], ExtremeNet [29], RepPoints [30], and YOLOX [23]
- Anchor-point-based algorithms directly predict the center point of the object and perform object bounding box regression, such as FSAF [31], FCOS [32], FoveaBox [33], and SAPD [34]

However, the anchor-based algorithms have some shortcomings for small object detection.

1. The imbalance of positive and negative samples: Anchors are usually sampled on the feature maps, while for pictures of small objects, most areas are background. It leads to a large number of simple negative samples, which have no useful effect on the network.
2. It is difficult to adjust to hyperparameters: Multiple hyperparameters of the anchor, such as number, size, width, and height, should be designed according to the actual situation and datasets. For small objects, if the anchor is too big, the IoU loss between the anchor and the ground truth will be too large, leading to no positive samples.
3. Anchor matching takes serious time in the training: To determine whether each anchor is a positive sample or a negative sample, it will calculate the IoU losses between each anchor and all ground truths, which will occupy many memory resources and will consume more calculation time.

The anchor-free method greatly reduces the number of parameters needing manual design and many skills involved and achieves good results in small object detection. Therefore, this paper adopts the YOLOX model based on the anchor-free method as the basic model for research.

## 3. Method

### 3.1. Architecture

**Figure 1:**
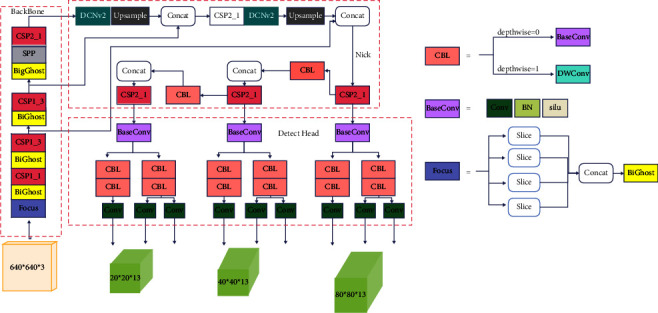
The architecture of BGD-YOLOX. ★ represents the modified part of YOLOX.

The one-stage object detection network is usually composed of the backbone network for feature extraction, the detection neck for feature fusion, and the detection head for classification and regression. To improve the detection performance of YOLOX on small objects in terms of network structure, we first modify the DarkNet53 backbone network of YOLOX as the backbone network studied in this paper, and then, we optimize the detection neck to enhance the feature extraction ability. The overall architecture of the improved YOLOX model (BGD-YOLOX) is shown in Figure 1.

#### 3.1.1. Backbone

**Figure 2:**
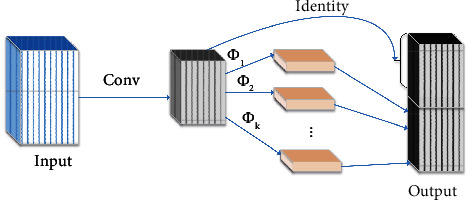
Ghost model. Conv represents convolution, *ϕ*_*i*_ represents linear operation, and identity represents identity mapping.

The YOLOX backbone network is DarkNet53, used to extract feature maps of different scales. Ding et al. [35] proposed that convolution with a large kernel is more conducive to downstream tasks such as object detection and semantic segmentation and is still effective on small feature maps. The sizeable effective receptive fields (ERF) can be constructed via large kernels [36]. Moreover, large convolutions leverage more shape information than traditional CNN and are more consistent with human cognition. However, the simple use of large kernel convolutions will greatly increase the cost of convolution. GhostNet [37] proposed the Ghost module, which replaces the traditional convolution with a simpler linear operation (depth-wise convolution) and generates redundant feature maps to reduce the number of parameters and computations and improve the network performance. The Ghost module is shown in Figure 2.

Let the number of input channels be *m*, the number of linear operations be *s*, where the last *ϕ*_i_ is the identity map used to retain the original feature map, and the number of output channels be n. There is actually one identity mapping and *m* × (*s* − 1)=*n*/*s* × (*s* − 1) linear operations. The supposed convolution kernel size of each linear operation is d × d, and in the case that the number of input channels *c* is far greater than the number of linear operations *s*, the acceleration ratio *r*_*p*_ of using the Ghost module instead of traditional convolution is

(1) $$\begin{array}{c} r_{p}=\frac{n\times h^{1}\times w^{1}\times c\times k\times k}{n/s\times h^{1}\times w^{1}\times c\times k^{2}+n/s\times \left(s-1\right)\times h^{1}\times w^{1}\times c\times d^{2}}\approx \frac{s\times c}{s+c-1}\approx s\text{.} \end{array}$$

Using the Ghost module instead of traditional convolution can reduce the number of parameters and computations of the network and prune the model.

**Figure 3:**
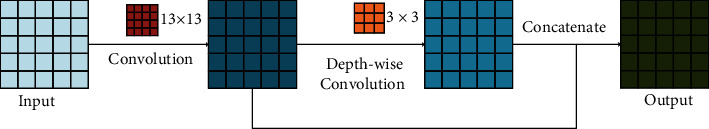
BigGhost model.

To enhance the network's performance of small object detection, we propose a BigGhost module based on large kernel convolution [35] and Ghost module [37], replacing some convolution layers of the original DarkNet53 backbone network. The BigGhost module uses the Ghost module to replace the ordinary convolution and joins the idea of the large kernel convolution at the same time. The structure of the BigGhost module is shown in Figure 3. We first use the 13 × 13 traditional convolution, and the number of channels is half of the number of output channels. Then, we apply a 3 × 3 depth-wise convolution, with the number of channels as half of the output channels. Finally, the results of the two convolutions are concatenated to obtain the final output feature map.

#### 3.1.2. Detection Neck

FPN [12] and PAN [16] are often used as the detection necks of object detection networks to construct feature pyramids. It connects horizontally between feature maps and carries out feature fusion top-down or bottom-up [38, 39]. The detection neck of YOLOX is a FAN + PAN bidirectional pyramid structure that is the same as YOLOv5. To improve the feature fusion result of the detected neck for small objects and obtain more key feature information, this study tries to add the modulated deformable convolution [40] to the detection neck. We replace two conventional convolutions in the PAN structure of the neck with two modulated deformable convolutions, which can enhance the feature extraction ability of the convolutions in the bottom-up feature fusion process [41, 42].

**Figure 4:**
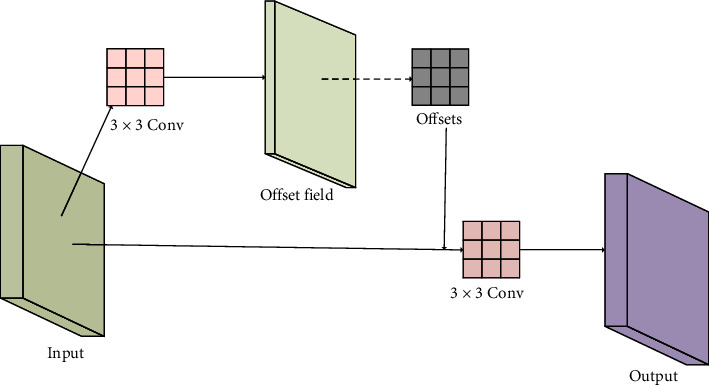
The structure of modulated deformable convolution. Conv represents convolution.

The effectiveness of the deformable convolutional network (DCN) [36] has been verified by many object detection algorithms. Deformable convolution can study various convolution kernel shapes according to different data and enable the model to learn the offsets of the sampling points of the convolution kernel on the input feature map. DCNv2 [40] proposed the modulated deformable convolution at the base of DCN. In addition to learning the offsets of the sampling points of the convolution kernel, the modulated deformable convolution also learns the weight of each sampling point to reduce the interference of irrelevant factors. Its network structure is the same as that of the deformable convolution, except for adding one parameter to represent the weight of sampling points. As shown in Figure 4, the modulated deformable convolution has two parts:

1. Using convolution to generate the offsets of the convolution kernel sampling points along the *x* and *y* directions on the input feature map
2. Based on the input feature map and the calculated offsets, we carry out bilinear interpolation and obtain the position of the sampling point of the convolution kernel on the input feature maps, and finally, we perform convolution

The modulated deformable convolution itself will not significantly increase the number of parameters and computations in the model. However, in practice, too many modulated deformable convolution layers will greatly raise the computation time. Therefore, to balance the efficiency and validity, we replace the two 3 × 3 convolution layers of the PAN with a modulated deformable convolution in DCNv2.

The outputs of the YOLOX's backbone network are inputs of the detected neck. The input features are fused by the bidirectional feature pyramid. The outputs of the neck are the output features with three different resolutions generated in the bottom-up fusion process of the PAN. Finally, we send them to the detection head for prediction. The details of the detection neck layer are shown in Figure 1.

#### 3.1.3. Detection Head

The detection head is shown in Figure 1. YOLOX uses the decoupled head to make multiscale predictions, which greatly improved the model convergence speed. Decoupling the detection head will increase the computational complexity. Therefore, it uses a 1 × 1 convolutional layer to reduce the channel dimension. Then, it is followed by two parallel branches with two 3 × 3 convolutional layers for class prediction and regression prediction, respectively. The regression branch is composed of two paratactic branches for bounding box prediction and confidence prediction. Binary cross-entropy (BCE) loss is used to train the class prediction branch and confidence prediction branch, and IoU loss is used to train bounding box prediction branches.

### 3.2. Training Tricks

#### 3.2.1. EMA

This paper adopts the exponential moving average (EMA) optimization strategy. EMA is an averaging method that gives a higher weight to recent data and helps control the moving average of training parameters. The value obtained by the moving average is gentler and smoother on the image, and the jitter is less. The moving average will not fluctuate greatly due to a certain abnormal value. EMA trains the parameters by using exponential decay to calculate moving averages. For each parameter, a shadow parameter is maintained:

(2) $$\begin{array}{c} W_{EMA}=\lambda W_{EMA}+\left(1-W_{EMA}\right)W\text{,} \end{array}$$

where *λ* is the decay rate. We apply EMA with a decay rate of 0.9998 in the experiment and use the shadow parameter *W*_EMA_ for evaluation.

#### 3.2.2. Data Augmentation

As the same as YOLOX, we use Mosaic [1] and Mixup [43] data augmentation during training and turn it off at the last 15 epochs to prevent overfitting. Mosaic data augmentation improves the network's ability to detect small objects by randomly cropping, scaling, rotating, and then stitching multiple images together. Mixup data augmentation randomly overlaps and mixes various images proportionally, which can enhance the linear expression ability between training samples and improve the generalization ability of the network.

#### 3.2.3. SimOTA

In terms of the label assignment strategy, YOLOX simplified OTA [44] and named it SimOTA. It automatically analyzes how many positive samples each ground truth should have and determines which feature maps to detect each ground truth. SimOTA first calculates the pairwise matching of ground truth and prediction, expressed as the cost of each ground truth and prediction pair. The cost between the ground truth *g*_*i*_ and prediction *p*_*j*_ is

(3) $$\begin{array}{c} c_{ij}=L_{ij}^{cls}+\lambda L_{ij}^{reg}\text{,} \end{array}$$

where *λ* is the equilibrium coefficient. *L*_*ij*_^cls^ and *L*_*ij*_^reg^ are the classification loss and regression loss between the ground truth *g*_*i*_ and prediction *p*_*j*_. Then, for the ground truth, the first *k* predictions with the least cost in the fixed center area will be selected as their positive samples and the rest as their negative samples.

## 4. Experiment

We justify the significance of BGD-YOLOX by a series of ablation studies and comparisons and verify the generalization performance on small object detection.

### 4.1. Experiments Settings

We used Windows 10 operating system, NVIDIA GeForce RTX 3060 12 GB GPU for calculation. The PyTorch framework is used. The torch version is 1.9.0, the CUDA version is 11.3, and the Python version is Python3.8.

YOLOX has standard models such as YOLOX_S, YOLOX_M, YOLOX_L, and YOLOX_X, whose networks are all the same, but the model sizes are different due to different numbers of layers. Therefore, we used the YOLOX_S version for all the following experiments.

During training, we resize the input images to 640 × 640 resolution. We use a global batch size of 8, SiLU activation function, and EMA strategy. What is more, we use standard SGD with a momentum coefficient of 0.9 and weight decay of 0.0005. We choose Warmup [45] + Cosine learning rate annealing [46] to adjust the learning rate, and the learning rate initialized as 0.1. Mosaic and mixup data augmentation probabilities are initialized as 1, and we turn off data augmentation at the last 15 epochs. All the models are trained from scratch for 300 epochs with the same simple training settings described previously.

### 4.2. Dataset

We verify the generalization performance of BGD-YOLOX for small object detection on RSOD [47] (Table 1), an open small object detection dataset. The dataset is randomly sampled at a ratio of 8 : 2 as the training set and test set of experiments. The dataset includes four types of remote sensing images and VOC labels: aircraft, oil tank, playground, and overpass, which are characterized by small pixels of detection objects, varied image scales, and complex backgrounds [47].

### 4.3. Ablation Studies

In this subsection, we verify the significance of our BGD-YOLOX (Table 2). We performed ablation studies to demonstrate the effectiveness of each part. Each part of the optimization is not completely independent; some optimization techniques are ineffective when used alone but effective when combined. Therefore, we show how to gradually improve the performance of our object detector in order to verify the effectiveness of the optimized parts.

*A* ⟶ *C*. First, based on the original YOLOX (*A*), we try to directly replace the 3 × 3 conv layers of the backbone network with the 13 × 13 big convolution to obtain model *B*. The 13 × 13 big convolution is represented by BigConv. The mAP0.5 is the mAP (mean average precision) when the IoU threshold is 0.5. The mAP0.95 represents the average mAP at different IoU thresholds, which change from 0.5 to 0.95 with a step size of 0.05. The mAP0.5 and mAP0.95 of model B are 85.1% and 57.0%, respectively, significantly improved compared with the original YOLOX (*A*), which verifies the effectiveness of the large kernel convolution mentioned in [35] for downstream tasks such as object detection. However, we find that the increase of the convolution kernel will lead to the doubling of the parameters and GFLOPs and affect the reference speed. Therefore, to further reduce the parameters and computations, we may optionally replace the 3 × 3 conv layers of the original YOLOX (*A*) backbone network with the BigGhost modules to trade accuracy for efficiency (*C*). Compared with the original YOLOX (*A*), the mAP0.5 of C is 84.1%, higher by 0.5%, and the mAP0.95 of *C* is 54.0%, lower by 0.2%. Compared with model B, mAP0.5 and mAP0.95 of model *C* decreased slightly, but the increase in parameters, computations, and inference time decreased by more than half on the basis of model *A*.

*A* ⟶ *D*. Then, we try to add modulated deformable convolution to the detected neck of the original YOLOX (*A*) model and obtain model *D*. The modulated deformable convolution is represented by DCNv2. Compared with the original YOLOX (*A*), the parameters increased by 0.09 M and the number of GFLOPs increased by 0.15 G, which is very small compared with that of the whole model. MAP0.95 increases by 1.7%, while mAP0.5 merely decreases by 1.2%.

*D* ⟶ *E*. From the previous experiments, we found that the effect of adding BigGhost and DCNv2 to the original YOLOX (*A*) is inconspicuous. However, when we try to add BigGhost and DCNv2 to the network at the same time, to get our model BGD-YOLOX(E), the number of parameters and GFLOPs almost has no increase compared to BigGhost alone, but the effect is dramatically improved. In comparison with the original model YOLOX (*A*), mAP0.5 directly improves by 4.7%, and mAP0.95 outperforms by 2.5%. We can infer that the modulated deformable convolution is more suitable for feature maps with more details. In other words, it is better to use it after large kernel convolutions.

**Figure 5:**
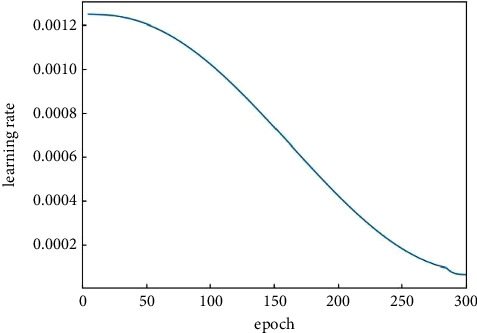
The curve of the learning rate.

**Figure 6:**
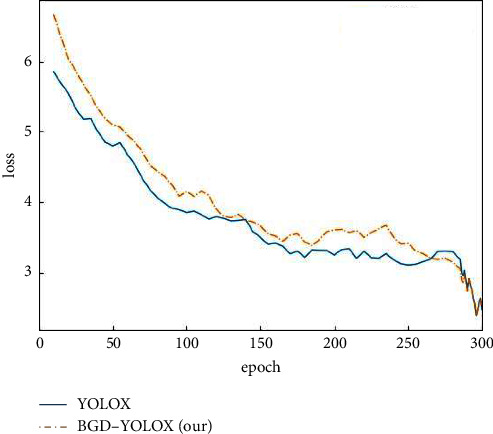
The loss curves of YOLOX and BGD-YOLOX.

**Figure 7:**
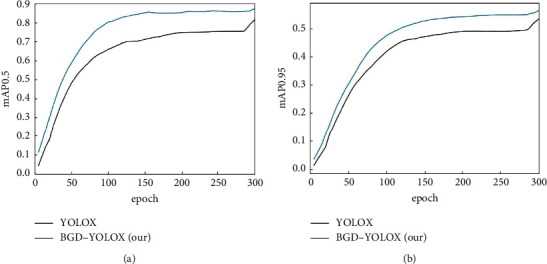
The mAP curves of YOLOX and BGD-YOLOX. (a) mAP0.5. (b) mAP0.95.

A good learning rate will raise the loss to fall to the lowest value faster and ensure it is the global optimal value. The adaptive learning rate changing curve (Figure 5) is adjusted by the warmup and cosine annealing mechanism. We perform the previous ablation studies while keeping other settings identical, so the learning rate curves of all experiments are the same. Figures 6 and 7 show the loss curves and mAP curves of YOLOX and BGD-YOLOX. It can be precisely shown from Figure 6 that the loss value gradually decreases with the increase of epochs and finally reaches the minimum value. By comparison, the loss curve of improved BGD-YOLOX is smoother. With the increase of epochs, the loss value gradually decreases and is lower than YOLOX finally. As shown in Figure 7, the mAP0.5 and mAP0.95 of BGD-YOLOX are always higher than those of YOLOX at the beginning of training, and the gap between them gradually increases with epochs. Furthermore, with the data augmentation turned off at the final 15 epochs, mAP0.5 and mAP0.95 of BGD-YOLOX slightly increase again. It also verifies again that excessive data augmentation will affect performance as mentioned in [23]. At the last epochs of training, turning off all data augmentation techniques may improve the detector's performance.

**Figure 8:**
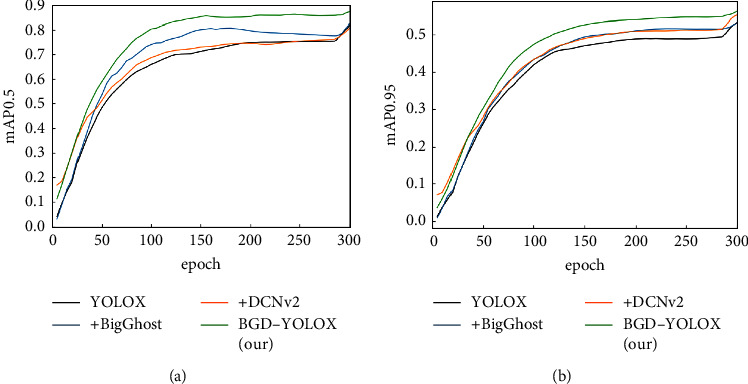
The mAP curves of ablation studies. (a) mAP0.5. (b) mAP0.95.

**Figure 9:**
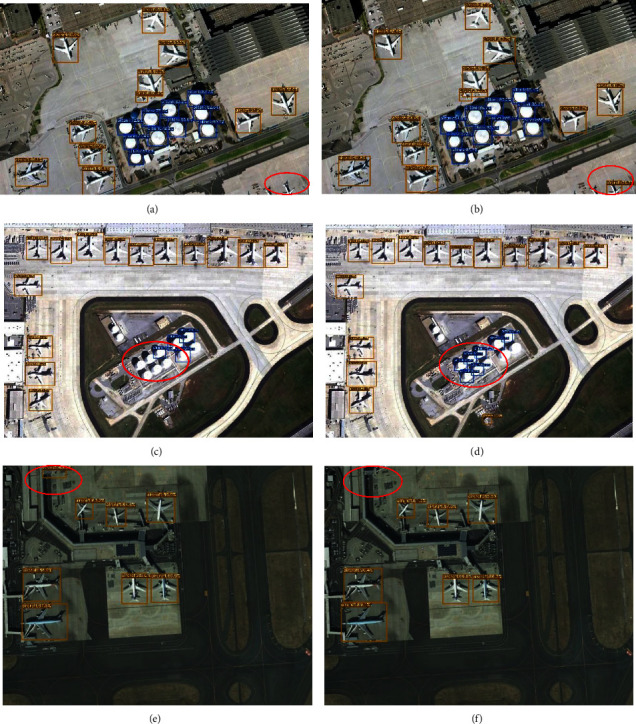
Comparisons of the detection effect; (a), (c), and (e) are the YOLOX detection effect; (b), (d), and (f) are the BGD-YOLOX detection effect.

To verify the effectiveness of our model optimization more intuitively, we draw the mAP curves of each part of the optimization network. MAP0.5 and mAP0.95 are experimental measurement indexes. The results are shown in Figure 8. It is obvious that when BigGhost and DCNv2 are used together, the performance is much higher than the effect of using them alone. MAP0.5 and mAP0.95 improve significantly. The comparison of the detection effects of BGD-YOLOX and original YOLOX on small objects is shown in Figure 9. We can see that the accuracy of BGD-YOLOX is higher than the initial model, and the probability of missed detection and error detection is lower.

### 4.4. Comparisons

Compared with the state-of-the-art detectors, BGD-YOLOX also shows favorable performance (Table 3). For a fair comparison, we train all the models on the same GPU. All the models are trained from scratch for 300 epochs with the same simple training settings described previously. By comparison with other state-of-the-art methods, our BGD-YOLOX has certain advantages in terms of parameters, computations, and precision. For example, contrasted with YOLOv4, the mAP0.5 of BGD-YOLO is 13.1% higher and the mAP0.95 of BGD-YOLO is 18.3% higher. At the same time, the number of parameters of BGD-YOLOX is about 1/3 of YOLOv4. Compared with other object detectors, BGD-YOLOX has advantages in precision, parameters, and computations in small object detection.

## 5. Conclusions

This study proposes BGD-YOLOX, a small object detection algorithm based on large kernel convolution and modulated deformable convolution, which reaches over 88% mAP on the RSOD dataset and shows favorable performance in small object detection compared to the state-of-the-art models, such as EfficientDet, Faster R-CNN, and YOLOv4. Specifically, we presented the BigGhost module and combined it with a modulated deformable convolution to improve the detection performance of small objects based on the YOLOX model and verified its effectiveness through a series of ablation studies and comparisons. The BGD-YOLOX model proposed in this paper has better performance in small object detection, with a lower miss rate and error rate but a higher precision.

## Figures and Tables

**Table 1:** The information of the RSOD dataset.

Classes	Pictures	Objects
Aircraft	446	4993
Playground	189	191
Overpass	176	180
Oil tank	165	1586

**Table 2:** The ablation study results.

	Methods	Parameters (M)	GFLOPs	mAP0.5 (%)	mAP0.95 (%)	Infer time (ms)
*A*	YOLOX	8.94	26.64	83.6	54.2	8.60
*B*	*A* + BigConv	36.85	106.33	85.1	57.0	13.16
*C*	*A* + BigGhost	21.46	63.78	84.1	54.0	10.88
*D*	*A* + DCNv2	9.03	26.79	82.4	55.9	9.73
*E*	*D* + BigGhost	21.55	63.93	88.3	56.7	12.84

**Table 3:** Comparisons of the speed and accuracy of different object detectors.

Methods	mAP0.5 (%)	mAP0.95 (%)	Parameters (M)	GFLOPs
YOLOX	83.6	54.2	8.94	26.64
YOLOv3 [3]	70.8	33.6	61.54	65.54
YOLOv4 [1]	75.2	38.4	64.36	60.33
EfficientDet-d0 [18]	54.4	22.7	3.83	4.61
RetinaNet [7]	85.1	47.8	36.39	146.00
Faster R-CNN [24]	67.5	34.0	28.31	939.45
CenterNet [26]	87.9	51.6	32.67	109.34
SSD [19]	65.0	30.2	24.01	61.11
BGD-YOLOX (ours)	88.3	56.7	21.55	63.93

## Data Availability

The data used to support this study can be found at https://github.com/RSIA-LIESMARS-WHU/RSOD-Dataset-.
